# Pneumonia, with Septic Lepto-Meningitis

**Published:** 1887-12

**Authors:** 


### CLINICAL NOTES.

### PNEUMONIA, WITH SEPTIC LEPTO-MENINGITIS.

[From Cook County Hospital. Reported by the Internes.]

### CASE I.

F—— B——, aet., 23 years; occupation, laborer. Had been sick 11 days before admission to the hospital. Admitted Nov. 7th. Died Nov. 14th.

No family history. No venereal history. Has been a dock hand, and confesses to have been a moderate drinker. Was sick in St. Louis two years ago with intermittent fever, and has not been well since then, suffering from time to time with neuralgia and dyspepsia. His present illness commenced 11 days ago with sudden and severe pain through head and chest. Following that, for three days, were severe rigors, followed by fever, vomiting and exacerbations of the headache. Since his illness commenced he has suffered from entire loss of sleep and from constipation, his bowels not having moved for four days.

Examination of the patient revealed equally contracted pupils, no paralysis of the extremities, retracted abdomen, with slight amount of gurgling in both *iliac* fossæ; the tongue and lips were dry, harsh and coated; there was cutaneous hyperæsthesia strongly marked, especially over abdomen and thorax; the temperature on admission was 103° F.; respiration 32; pulse 84, and full; urinalysis showed a specific gravity of 1032, with a trace of albumen, but no sediment. The patient lay in a stupor, from which he could be easily aroused to intelligence, but always with an uneasy expression of pain. When aroused he would immediately complain of great pain in the head. He had also a short, dry cough of an apparently nervous origin. Any attempt at a physical exploration of the thorax was resisted by the patient with renewed complaint of pain.

Probable diagnosis: Acute lepto-meningitis. Treatment: Ice cap to head. Antipyrin, grs. xv. when the temperature exceeded 102°. Bromide and iodide of potassium in moderate doses at first, then pushed. Fluid extract of ergot in ℥ss. doses. Magendi's solution p. r. n. to relieve pain. Cathartics, at first mild, then enemata and fortified oil (ol. ricini ℥i, ol. tiglii gtt. i.) when bowels failed to respond to gentler measures. The progress of the case was unfavorable. The range of temperature was from 99° to 105°; the latter was reached just before death. No evening exacerbation. The pulse ranged between 90 and 120, becoming imperceptible at the last. Respiration ranged from 30 on admission to 64 just before death, and was labored, with marked stertor during the latter days. During the night there was at first muttering, delirium, and at times stupor. Toward the last he became comatose. The pupils were dilated before death. There were involuntary evacuations of rectum and bladder for some time preceding the termination of the case. No paralysis of motion or sensation was observed except in parts supplied by the cranial nerves. He moved aimlessly from time to time, even just before death.

Post-mortem 20 hours after death. Rigor decided. Abdomen retracted. No external markings. Body emaciated. Heart normal.

Lungs: Adhesions over both apices. Lower lobe of left lung is in a condition of red hepatization, with small insular patches of gray hepatization commencing.

Spleen: Liver and kidneys are a trifle soft and swollen.

Stomach and intestines not opened.

Head: Sinuses of the dura mater are more than usually full, but without thrombi. On opening the dura the convolutions of the cerebrum are seen to be more than usually flattened against the dura. There is no excess of fluid in the sub-dural or sub-arachnoid spaces. The pia mater of the convexity is deeply congested, and in the larger sulci there is a large amount of fibrinous effusion. On examining the base of the brain the following appearances were observed: For a distance of 8 cm. from the optic chiasma backwards, below medulla, and between the bases of the cerebellar lobes, and extending in width 3 cm. either side of the median line, there is creamy, semi-solid exudate, involving the infundibulum, perforated spaces, optic tracts, cerebral peduncles, pons, medulla, and the superficial roots of all the cranial nerves. Exudate is entirely beneath the pia, not involving the dura or nerve sheaths, or the bony structures of the base of the skull.

The base of the left middle lobe is soft to the touch, and fluctuation can be elicited at any point over the area of softness.

Incision through corpus callosum into right lateral ventricle discloses a small amount of slightly turbid fluid, not distinctly purulent. Brain substance is not softened.

Incision into left lateral ventricle shows the ventricle to be enlarged and filled with thick greenish pus, similar to that noticed at the base. The exudate communicates through the infundibulum with that at the base. The choroid plexus is covered with pus and the walls of ventricle are softened. The spot of softening felt from without is found to be due to the extension of the softening and suppuration from the ventricle into the brain substance. Both basal ganglia are soft. The third and fourth ventricles are filled with pus and the walls are softened.

Microscopical examination reveals copious exudation of leucocytes into pia mater and into gray and white substance of all structures involved.

